# CRISPR in mobile genetic elements: counter-defense, inter-element competition and RNA-guided transposition

**DOI:** 10.1186/s12915-024-02090-x

**Published:** 2024-12-18

**Authors:** Eugene V. Koonin, Kira S. Makarova

**Affiliations:** Computational Biology Branch, Division of Intramural Research, National Library of Medicine, National Institutes of Health, Bethesda, MD 20894 USA

**Keywords:** CRISPR systems, CRISPR-associated transposases, Mobile genetic elements, Exaptation

## Abstract

CRISPR are adaptive immunity systems that protect bacteria and archaea from viruses and other mobile genetic elements (MGE) via an RNA-guided interference mechanism. However, in the course of the host-parasite co-evolution, CRISPR systems have been recruited by MGE themselves for counter-defense or other functions. Some bacteriophages encode fully functional CRISPR systems that target host defense systems, and many others recruited individual components of CRISPR systems, such as single repeat units that inhibit host CRISPR systems and CRISPR mini-arrays that target related viruses contributing to inter-virus competition. Many plasmids carry type IV or subtype V-M CRISPR systems that appear to be involved in inter-plasmid competition. Numerous Tn7-like and Mu-like transposons encode CRISPR-associated transposases (CASTs) in which interference-defective CRISPR systems of type I or type V mediate RNA-guided, site-specific transposition. The recruitment of CRISPR systems and their components by MGE is a manifestation of extensive gene shuttling between host immune systems and MGE, a major trend in the coevolution of MGE with their hosts.

## Background

CRISPR-Cas (CRISPR for short) are bacterial and archaeal adaptive immune systems that consist of an array of direct repeats separated by unique spacers and the adjacent CRISPR-associated (*cas*) genes [1–3]. Cas1 and Cas2 proteins, in some CRISPR subtypes assisted by additional proteins, constitute the CRISPR adaptation (spacer acquisition) module that inserts a fragment of foreign (in particular, viral) DNA between the proximal repeats in the array, yielding a spacer complementary to a segment of the target genome (protospacer), which is accompanied by repeat duplication. Transcripts of the CRISPR array are processed into small, unit size CRISPR (cr) RNAs by a distinct complex of Cas proteins or an external enzyme and serve as guides for highly specific recognition of the target DNA or RNA that is typically followed by the target cleavage by a dedicated Cas nuclease. Characteristically of defense systems that evolve in a perennial arms race with mobile genetic elements (MGE), CRISPR show pronounced diversity, with two classes, 7 types, and numerous subtypes that differ with regard to the composition of Cas proteins and the organization of the CRISPR-*cas* genomic loci [4, 5]. In particular, each of the 7 CRISPR types encompasses a distinct effector nuclease.

In 2018, one of the authors of this review published a BMC Biology Editorial on open questions in CRISPR biology [6]. Altogether, 10 such unresolved problems have been formulated, dealing primarily with the distribution of CRISPR systems across the diversity of archaea and bacteria, the biological roles of different types of CRISPR, and their evolution. It appears that these questions were indeed quite challenging because 6 years after the publication, most of them remain as open as they were when first asked. However, on one of these problems, number 9 on the list, “What are the functions of CRISPR-Cas systems encoded by transposons and plasmids?”, the progress has been impressive. Thus, here, we focus on the recruitment of CRISPR systems and their components by mobile genetic elements (MGE) including viruses and their functional repurposing (exaptation).

Almost all life forms harbor multiple, diverse MGE, some of which integrate into the host genome, whereas others replicate as free elements [7, 8]. The three main classes of MGEs are transposons, plasmids, and viruses which employ substantially different mechanisms of mobility. Remarkably, in prokaryotes, members of all these three types of MGE have on multiple occasions recruited CRISPR systems or their components that in some cases retain their full functionality but more commonly, lose some of their activities and are repurposed for distinct roles in MGE reproduction.

In this brief review article, we discuss the spread and diversity of CRISPR systems and their components in MGE, modifications associated with their recruitment, and the latest findings on their functions in MGE reproduction.

## Main text

### CRISPR in viruses: counter-defense and inter-virus competition

**Fig. 1:**
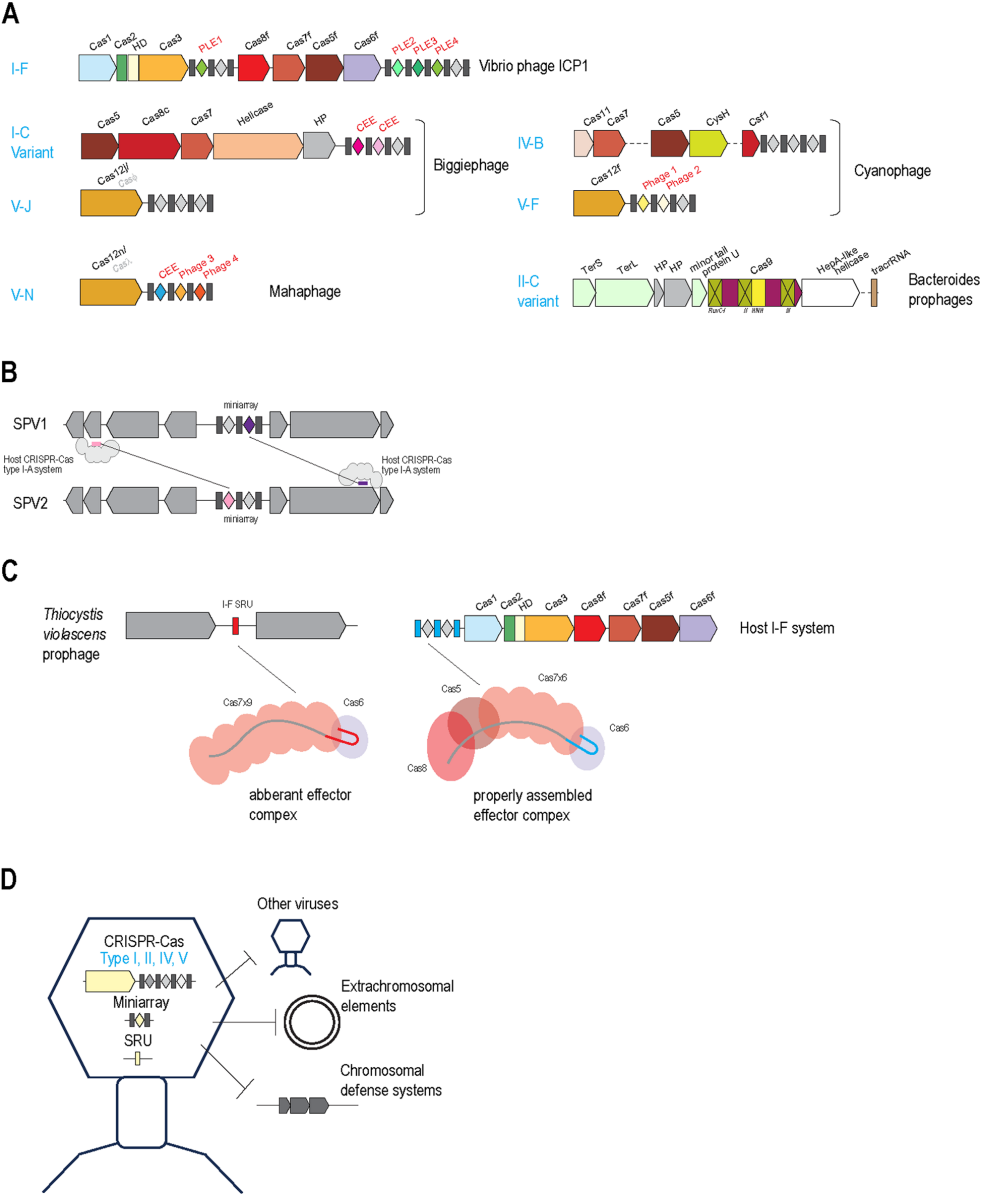
CRISPR systems and their components encoded in viral genomes. **A** Organization of the CRISPR loci carried by viruses. Genes are shown by block arrows, roughly to scale. CRISPR repeats are shown by rectangles, and spacers are shown by diamonds; spacers with detectable matches are shown with colors, and spacers without matches are shown in grey. *Cas* genes are denoted by their systematic names, with some legacy names shown in grey. CEE, circular extrachromosomal element; HD, nuclease of HD family (named for its catalytic dyad); HP; hypothetical protein; PLE, phage-like element; TerS (terminase small subunit), TerL (terminase large subunit) and phage minor tail protein U, essential phage proteins; tracrRNA, transactivating CRISPR RNA. For the phage-encoded Cas9c protein, inactivation of the three catalytic motifs of the RuvC-like nuclease is indicated. **B** Competition between two archaeal viruses mediated by viral CRISPR mini-arrays; Spacers with matching protospacers in the respective viral genomes are shown in black and maroon, and spacers without matches are shown in grey. SPV1, SPV2, Saccharolobus portogloboviruses 1 and 2, two closely related, non-lytic archaeal viruses [14]. The other designations are as in **A**. **C** Inhibition of a host CRISPR systems by virus-encoded RNA anti-CRISPR. SRU, single repeat unit. Other designations are as in **A**. **D** General schematic of the functions of virus-encoded CRISPR systems and CRISPR components

The study of CRISPR recruitment by MGE started in the now classic 2013 study by Seed and colleagues on the type I-F CRISPR encoded by the Vibrio phage ICP1 [9]. This phage-encoded CRISPR system is fully functional, complete with a CRISPR array and adaptation module (Fig. 1A), and contains multiple spacers that target a *Vibrio* antiphage-defense module, phage-induced island-like element (PLE), enabling ICP1 reproduction. Notably, this CRISPR system is only present in a subset of ICP1 strains where it occupies a specific antidefense locus that in other strains is occupied by the GIY-YIG family nuclease Odn which inhibits PLE via a completely different mechanism [10, 11]. The type I-F CRISPR system encoded by ICP1 is unrelated to the I-E system encoded by the host bacterium, so it remains unclear where, when, and how ICP1 captured the CRISPR locus.

ISP1 currently remains the only CRISPR-encoding phage for which the antidefense activity of the CRISPR system has been studied experimentally. Phage genome surveys show that CRISPR systems are rarely encoded by phages in the typical genome size range (below 200 kb) [12]. By contrast, analysis of the large (> 200 kb) genomes of jumbo phages assembled from metagenomic sequences led to the identification of numerous CRISPR systems of different types some of which appear to be fully functional whereas others lack either the adaptation module or the nuclease required for interference, or both (Fig. 1A) [13].

A broader variety of phages and archaeal viruses encompass minimal CRISPR components, namely, CRISPR mini-arrays (that is, arrays including only one or two spacers) and single repeat units (Fig. 1B, C) [12]. The mini-arrays contain repeats identical to those in the CRISPR systems of the respective hosts, and the majority of the spacers target viruses related to the given mini-array containing virus. This targeting specificity immediately prompted the hypothesis that the viral mini-arrays exploited the host CRISPR system to block replication of related viruses in coinfected host cells. This mechanism was validated in competition experiments with two related archaeal viruses one of which carried a CRISPR mini-array with a spacer against the other virus [14] (Fig. 1B).

The single CRISPR repeat units present in numerous viral genomes have been proposed to be transcribed into small RNAs mimicking CRISPR RNAs and acting as dominant-negative inhibitors of Cas proteins (RNA anti-CRISPR, Racr), forming non-productive effector complexes (Fig. 1C). Subsequently, it has been shown that a prophage-encoded Racr strongly inhibits the cognate type I-F CRISPR-Cas system by interacting specifically with Cas6f and Cas7f proteins, resulting in the formation of an aberrant Cas subcomplex [15]. In addition, Racrs have been shown to inhibit CRISPR systems of almost all other CRISPR types.

Altogether, virus-encoded CRISPR systems have been shown to contribute to all types of biological conflict in which viruses are involved including counter-defense, in particular, inactivation of defensive extrachromosomal elements, and intervirus competition (Fig. 1D).

Many viruses of bacteria and archaea encode diverse anti-CRISPR proteins (Acrs) [16, 17]. It would seem plausible that at least some of the Acrs would be Cas proteins or individual domains of these recruited by viruses and acting as dominant negative inhibitors of host CRISPR systems. Some complex viruses of eukaryotes, such as poxviruses, indeed have evolved multiple counter-defense proteins via this route [18]. Apparently, however, among the hundreds identified Acrs [19], very few were derived from Cas proteins. One of such rare cases includes homologs of the Cas4 nuclease encoded by some archaeal viruses one of which has been shown to inhibit CRISPR adaptation in model experiments [20]. Another example is AcrIII-1, an inhibitor of type III CRISPR systems which is a homolog of RING nucleases that cleaves cyclic oligoA, the second messenger synthesized by Cas10 protein, mitigating programmed cell death induction by these systems [21, 22]. Another notable case is the phage-encoded Cas9 protein with an inactivated RuvC-like nuclease domain that likely suppresses the host CRISPR immunity by sequestering crRNAs via the formation of stable complexes via a phage-encoded trans-activating CRISPR (tracr) RNA [5] (Fig. 1A). Finally, AcrIF3 appears to be a structural mimic of the domain of Cas8f protein (the large subunit of the Cascade complex) that recruits the Cas3 nuclease, and thus acts as a dominant-negative inhibitor of CRISPR interference [23]. These exceptions notwithstanding, it remains an enigma why segments of CRISPR RNAs (Racr) were broadly recruited by viruses as CRISPR inhibitors whereas Cas proteins apparently were not.

### CRISPR in plasmids: inter-plasmid competition and more

**Fig. 2:**
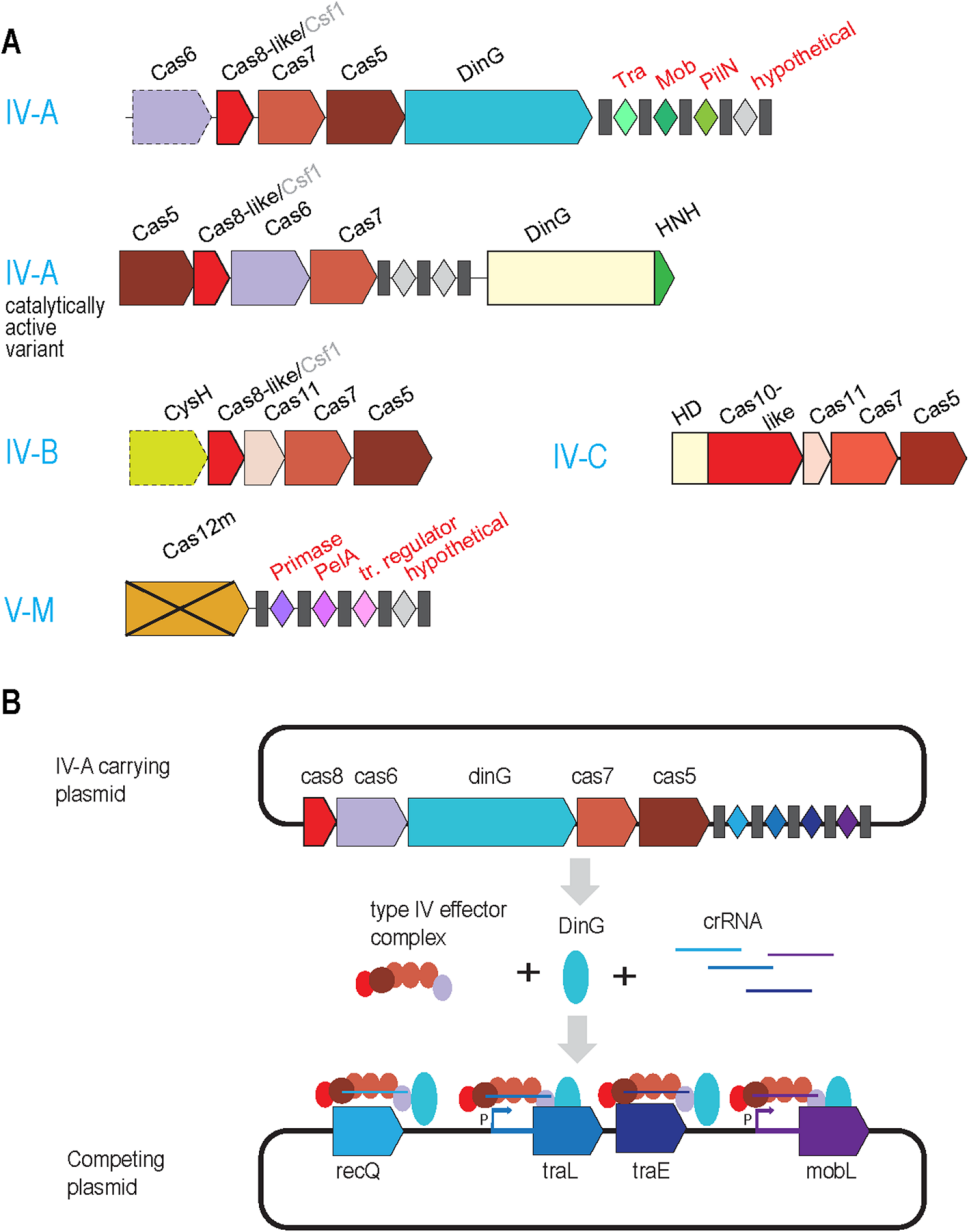
CRISPR systems carried by plasmids and their role in inter-plasmid competition. **A** Organization of the type IV and subtype V-M CRISPR loci carried by plasmids. Tra, Mob, PilN, and PelA are all essential plasmids genes; tr. regulator, transcription regulator. Block arrows with dashed outlines denote genes that are present in the majority but not all of the respective loci. Cas12m is an inactivated type V CRISPR nuclease. The other designations are as in Fig. 1. **B** Inter-plasmid competition mediated by a plasmid-encoded type IV CRISPR system via inhibition of transcription of essential plasmids genes. MobL, RecQ, TraE, and TraL are all essential plasmids proteins; P, promoter

Type IV CRISPR systems that appear to be derived descendants of type III systems are found primarily if not exclusively in plasmids, conjugative integrating elements, and some phages and prophages. Unlike typical Class 1 CRISPR systems, most of the type IV loci lack known or predicted effector nucleases, and in many cases, contain no adaptation module (Fig. 2A). Instead, subtype IV-A systems include a DinG helicase whereas subtype IV-B systems typically encompass a CysH-like protein, an inactivated derivative of phospho-adenylylsulphate reductase [24].

The CRISPR arrays in most type IV loci are strongly enriched with spacers targeting conjugative plasmids, suggesting a role in inter-plasmid competition [25–27]. Subsequently, it has been directly shown that, notwithstanding the lack of nuclease activity, plasmid-encoded subtype IV-A CRISPR can eliminate other plasmids from bacterial cells by crRNA-guided transcriptional repression of essential plasmid genes [28] (Fig. 2B). These findings were complemented by the demonstration that this plasmid-encoded CRISPR system can use the adaptation machinery of a host I-E CRISPR to incorporate spacers into its CRISPR array, compensating for the lack of an adaptation module in the type IV system [28]. Notably, one of the spacers in a type IV-A system from a *Pseudomonas oleovorans* megaplasmid targets host *pilN* gene, an essential component of type IV pili that are involved in DNA uptake, downregulating transcription of this gene [29]. In this case, the type IV system seems to contribute to plasmid competition indirectly, by preventing the uptake of other plasmids by a bacterium carrying the given one. Transcription inhibition might not be the only mechanism of interference by type IV-A CRISPR because they can block reproduction of target plasmids even via spacers matching non-coding regions [29, 30].

Although the most common type IV systems lack nucleases, in subtype IV-C, an HD nuclease domain is fused to the large subunit of the effector complex (Fig. 2). The presence of this fusion and the size of the large subunit, which is close to that of Cas10 and is in contrast to much smaller corresponding proteins in other type IV subtypes, suggests that IV-C systems are the evolutionary intermediate on the path from type III to type IV [27]. Furthermore, a recent large-scale search for novel CRISPR systems in metagenomes led to the identification of a rare subtype IV-A variant that contains an HNH nuclease domain fused to DinG (Fig. 2). The dsDNA target cleaving, spacer-dependent activity of this nuclease was demonstrated experimentally and was shown to depend on the helicase activity of DinG which moves along the target molecule unwinding dsDNA and allowing the HNH nuclease to shred the target [5]. This interference mechanism mimics the mechanism of type I CRISPR systems in which Cas3 helicase containing a fused HD nuclease domain unwinds and shreds the target. However, DinG is only distantly related to Cas3 indicating that the target-shredding helicase-nuclease fusion proteins evolved independently in type I and type IV CRISPR systems. Notably, some variants of subtype IV-B encode an even more distantly related helicase, a RecD homolog [27], emphasizing multiple convergent acquisitions of helicases by different CRISPR systems.

Subtype IV-B systems lack both the adaptation module and a CRISPR array which, together with the CysH association, suggest a distinct function in plasmids and integrating conjugative elements that might not involve target recognition and remains to be characterized.

In addition to plasmids, albeit less commonly, type IV CRISPR systems are encoded in some prophages, suggestive of roles in inter-phage or phage-plasmid competition [24, 27].

A systematic survey of CRISPR representation in plasmids showed that, apart from the plasmid-specific type IV, some plasmids encode CRISPR systems of all other types except for type VI, and subtypes III-B and V-F are specifically enriched in plasmids [31]. Particularly notable is subtype V-M that is strongly enriched in plasmids and also encoded by some prophages. The effector of this CRISPR subtype, Cas12m, contains an inactivated RuvC-like nuclease domain and has been shown to inhibit plasmid replication by inhibition of transcription of essential plasmid genes, without cleaving the target [32]. Similarly to the case of viruses (see above), CRISPR systems are most common in large (above 200 kb) compared to smaller plasmids. Predominant plasmid-targeting specificity was observed for spacers from most plasmid-encoded CRISPR systems suggesting that not only type IV, for which inter-plasmid competition appears to be the primary function, but other CRISPR systems recruited by plasmids contribute to this type of conflict. However, a substantial minority of spacers in plasmid-carried CRISPR loci targeted viruses indicating that, in some cases, these CRISPR systems protect the plasmid-carrying host from viruses, thus ensuring propagation of the plasmid.

### CASTs: recruiting CRISPR for RNA-guided transposition

**Fig. 3:**
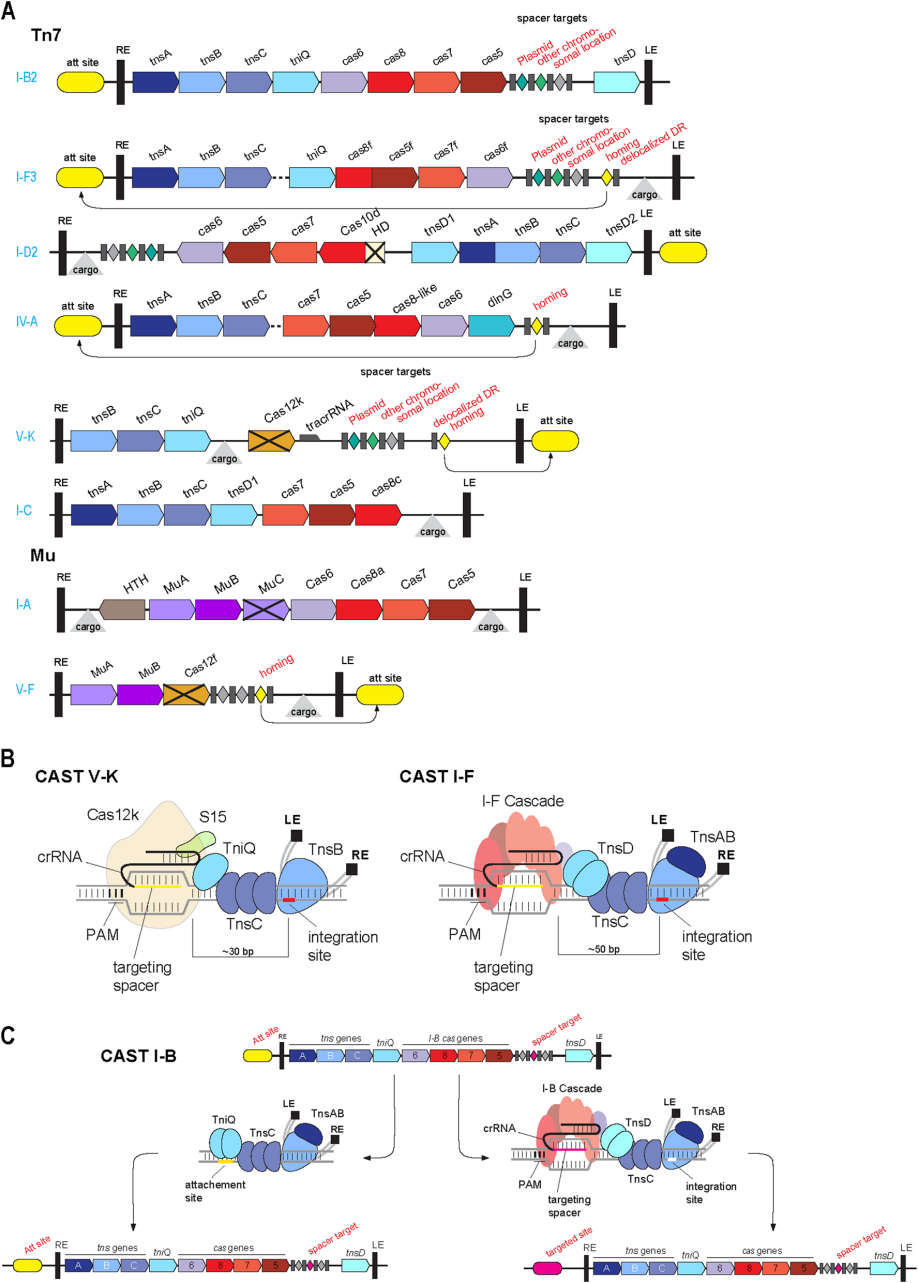
The CASTs: recruitment of CRISPR systems by Tn7-like and Mu-like transposons for RNA-guided transposition. **A** Organization of CAST-encoding Tn7-like and Mu-like transposons. *Tns* and *tni* genes encoded transposase subunits. *Att*, transposons attachment sites; HTH, helix-turn-helix DNA-binding protein; LE, RE, transposon left end and right end, respectively; DR, direct repeat. The designations are as in Fig. 1, but the genes are shown not to scale. Crossed block arrows denote inactivated enzymes. **B** Schematic depiction of the structures and functionalities of two distinct CASTs. PAM, protospacer-adjacent motifs. **C** The two alternative routes of CAST insertion. Genetic organization of the I-B CAST is shown. Underneath, on the left, is a schematic depiction of homing to the attachment site via TniQ, and on the right, a schematic depiction of RNA-guided transposition via TnsD root (on the right). At the bottom, are schematics of the new Tn7-carrying loci emerging as the result of the transposon insertion. LE, RE, transposon Left End and Right END, respectively

Comparative genomic analysis of the diversity of CRISPR systems led to the unexpected discovery of distinct type I and type V CRISPR variants embedded in several groups of Tn7-like transposons (hereafter, CAST, CRISPR-associated transposase) [33, 34]. Tn7-like transposons encompass diverse cargo genes, in addition to the multiple transposase subunits, and CASTs are among the most common types of cargo [35]. Most of the CASTs lack both the adaptation module and the effector nuclease, that is, they encompass the minimal complement of *cas* genes required for crRNA processing and target recognition, while losing the capacity for target cleavage (Fig. 3A). Initially, two independent cases of type I-B CRISPR capture and a single case of type I-F capture by Tn7-like transposons were discovered by phylogenetic analysis of both the Cas7 protein, the most highly conserved component of type I CRISPR systems, and TnsA and TnsD (TniQ), two subunits of the Tn7 transposase [33]. The type I-F CAST appears to have been an ancient acquisition because it is conserved in a large branch of Tn7-like transposons, whereas I-B CASTs are shared by small groups of transposons and thus were apparently acquired more recently. Additional cases of CRISPR capture by Tn7-like transposons were discovered by subsequent mining of genomic and metagenomic databases including subtypes I-C, I-E, I-D, IV-A, and V-K (Fig. 3A) [12, 36, 37]. Most of the type I CASTs have lost the Cas3 helicase-nuclease, and with it, the target cleavage capacity while retaining the subunits of the effector complex (known as CASCADE in type I CRISPR) involved in crRNA binding and target recognition. In contrast, in Cas12k, the single-protein effector of type V-K CRISPR, the interference capacity is abolished due to the replacement of individual catalytic residues in the RuvC-like nuclease domain. These distinct modes of inactivation point to strong selection driving the CASTs to lose the target-cleaving nuclease activity. Remarkably, in the case of CAST I-D, one variant lost Cas3 and contains a mutationally inactivated target-cleaving HD nuclease domain in Cas10d, whereas the other variant is a complete, functional CRISPR system, containing both the adaptation module and the effector nuclease [37, 38] (Fig. 3A). Clearly, the functional version of CAST I-D represents an early stage in CAST evolution [37, 38]. Deep mining of metagenomes for new CRISPR systems led to the discovery of I-A and V-F CASTs in Mu transposons, a family of phage-like transposons distantly related to Tn7, further supporting the notion that CRISPR systems have been recruited by cargo-carrying transposons on multiple, independent occasions [5] (Fig. 3A).

The discovery of the CASTs prompted the straightforward hypothesis that the minimal CRISPR systems recruited by Tn7-like transposons enabled RNA-guided transposition by targeting the transposons to unique genomic sites defined by the spacer sequences [33]. This hypothesis was promptly validated experimentally by demonstrating that CAST I-D, CAST I-F, and CAST V-K each formed complexes with the respective Tn7 transposases and, when programmed to recognize a unique site in the target DNA via an engineered spacer, mediated transposon insertion at a fixed distance of 30 to 50 bp away from the protospacer (Fig. 3B) [34, 39].

The structures of CAST I-F [40] and CAST V-K [41–44] transpososomes have been solved to atomic resolution by cryo-EM, revealing the details of the interactions between the Cas proteins and transposase subunit as well as target recognition. In CAST I-F, the Cascade complex is connected with the transposase via the dimer of the TniQ subunit, with one TniQ monomer interacting with Cas6 and the other one interacting with one of the 6 Cas7 subunits of the Cascade (Fig. 3B). In this system, the Cascade-TniQ complex scans numerous sites in the target DNA, but recruits TnsB and TnsC, the two transposase subunits, only to a few sites with sufficient complementarity to the spacer, suggesting a proofreading step [45]. In the CAST V-K transpososome, Cas12k protein, although structurally unrelated to Cascade, also interacts with TniQ which in turn recruits TnsC. Unexpectedly, the CAST V-K transpososome was found to include an additional subunit interacting with Cas12k, ribosomal protein S15 (Fig. 3B). In contrast to the other experimentally characterized CASTs, CAST I-D can mediate RNA-guided transposition even in the absence of TniQ, apparently, via direct interaction with TnsC, a core transposase subunit [37].

The role of the CASTs in the transposon life cycle remains incompletely understood. Tn7-like transposons propagate via two types of transposition, inserting either into variable sites in MGE (plasmids) or into conserved homing sites adjacent to tRNA genes or *glmS* genes in bacterial genomes (Fig. 3C) [46]. The very short CRISPR arrays associated with the CASTs contain some spacers targeting MGE, such as conjugative plasmids [37]. However, although the CASTs efficiently drive spacer-guided transposon insertion in model experiments and likely insert into MGE via the spacers from the array, this is not what happens during homing given that integrated copies of CASTs are not adjacent to protospacers. Instead, both CAST I-F and CAST V-K home to sites near tRNA or *glmS* genes through the so-called delocalized crRNA which is encoded near the CAST but outside of the array and contains sequences that only partially match the repeats in the array along with spacer-like sequences targeting short sequences adjacent to the homing sites [47, 48]. Furthermore, CAST variants containing only delocalized crRNA but no array have been discovered [37], suggesting that the respective transposons have lost the ability to integrate into MGE or integrate via a distinct mechanism independent of a guide RNA.

Whereas CAST I-F and CAST V-K apparently insert both into MGE and into conserved homing sites in bacterial genomes via the RNA-guided mechanism, CAST I-B employs two distinct mechanisms for these two types of transposition [48]. In this case, homing does not involve the Cascade or any guide RNA and is mediated by TnsD via a protein-only mechanism (Fig. 3C).

The intricacy and diversity of the interactions between the transposition machinery and Cas proteins and molecular mechanisms of the CASTs remain to be explored in detail. In particular, it is unclear how the CASTs acquire spacers and what is the origin of the delocalized crRNA. An unexpected functionality of the CASTs is suggested by the recent demonstration that CAST I-B, CAST I-F, and CAST V-K all can bind crRNAs from the host chromosomal CRISPR systems via a sequence-independent interaction between Cas6 and the repeat in the crRNA [49]. The spacer of the bound crRNA can then be used by the CAST for guided transposition. This could be an additional mechanism for integration of the CASTs into MGE given that spacers from the host arrays mostly target viruses and plasmids.

Last but not least, the potential of the CASTs for precise insertion of large pieces of DNA into bacterial and eukaryotic genomes is obvious. In spite of the technical difficulties due to the necessity of simultaneous expression of multiple proteins, the first successful experiments in this direction in bacterial [50–52] and human [53] models have been reported.

## Conclusions

CRISPR systems or their components have been recruited and repurposed by all major types of MGE in prokaryotes, viruses, plasmids, and transposons. There are three major types of functionality of CRISPR in MGE: (1) counter-defense, (2) inter-MGE competition, (3) RNA-guided transposition. The repeated co-option (exaptation) of RNA-guided target recognition machinery that originally evolved as an adaptive anti-MGE immunity mechanism to function in the propagation of MGEs themselves is a striking manifestation of the “guns for hire” principle, a major trend in the coevolution of MGE with their hosts whereby MGE extensively exchange components with host immune systems [54]. Indeed, recruitment of CRISPR by MGE discussed here is only one side of this relationship. At least two types of CRISPR systems, II and V, evolved from RNA-guided nucleases encoded by transposons of the IS200/605 family and involved in distinct modes of transposon propagation [55–59]. Furthermore, a distinct variety of prokaryotic transposons, the casposons, apparently gave rise to the CRISPR adaptation module [60]. Undoubtedly, further studies will reveal new connections between CRISPR and MGE and yield insights into the ecology of MGE-encoded CRISPR systems that currently remain poorly understood.

## Data Availability

No datasets were generated or analysed during the current study.

## Abbreviations

CAST: CRISPR associated transposase
CRISPR: Clustered regularly interspaced palindromic repeats
crRNA: CRISPR RNA
MGE: Mobile genetic element
tracrRNA: TRans-activating crRNA
